# Aftiphilin Regulation of Myosin Light Chain Kinase Activity Promotes Actin Dynamics and Intestinal Epithelial Barrier Function

**DOI:** 10.3389/fgstr.2022.901404

**Published:** 2022-05-27

**Authors:** Ivy Ka Man Law, Kai Fang, Charalabos Pothoulakis, Carl Robert Rankin

**Affiliations:** Vatche and Tamar Manoukian Division of Digestive Diseases, David Geffen School of Medicine, University of California Los Angeles (UCLA), Los Angeles, CA, United States

**Keywords:** aftiphilin, colonic epithelial cells, epithelial permeability, tight junction, actomyosin

## Abstract

The expression levels of aftiphilin (AFTPH) are significantly lower in inflamed colonic tissues from patients with ulcerative colitis (UC) and mice with experimental colitis. During colonic inflammation, the selective permeability of the colonic epithelium is compromised largely due to dysregulation of proteins associated with either the tight junction (TJ) complex and actomyosin contraction rings. Here, we hypothesized that inflammation-associated reduction in AFTPH levels might cause an increase in the selective permeability of the colonic epithelium. In this study, we measured the transepithelial electric resistance (TEER), sodium (Na^+^) ion flux and dextran permeability in polarized colonic epithelial cells after manipulation of AFTPH. Silencing of AFTPH reduced TEER, increased Na^+^ ion flow and dextran permeability. Examination of mRNA and protein levels of multiple TJ proteins and Na^+^ ion transporters suggested that AFTPH deficiency did not significantly change expression of most of these transmembrane proteins. While the gross structure of the TJs in AFTPH gene-silenced cells appeared normal, elevated levels of junctional Occludin were observed. Most notably we observed that AFTPH co-localized with myosin light chain kinase (MLCK) and attenuated cellular MLCK activity as observed by phospho- myosin light chain 2 (pMLC2) western blots. Importantly, inhibition of MLCK activity reversed the reduction of TEER in AFTPH-deficient monolayers. Lastly, examination of microvilli by transmission electron microscopy and immunofluorescence imaging of actin filament arrangement demonstrated that AFTPH deficiency also affected filament arrangement in colonic epithelial cells. Taken together, these results suggest that AFTPH regulates intestinal epithelial permeability and actin polymerization in colonic epithelium through interfering with MLCK/MLC interactions.

## Introduction

The intestinal barrier is formed by a single layer of polarized intestinal epithelial cells located between the underlying mucosa and lumen. At homeostasis, intestinal epithelial cells maintain a selective barrier, allowing the paracellular flux of ions, nutrients and water [review in (1)], through dynamic regulations of (i) tight junction (TJ) transmembrane proteins; (ii) contraction of actin-myosin rings; and (iii) ion transporters. At the boundary between apical and basolateral domains in intestinal epithelial cells, TJ transmembrane proteins connect between adjacent cells to create a functional gate for the paracellular flux of small ions and water (also known as the pore pathway. TJ transmembrane proteins can be grouped into three main families, claudins, TJ-associated MARVEL proteins (TAMPs), and the cortical thymocyte marker in *Xenopus* (CTX). Claudins are important in regulating paracellular, size-dependent, flux of charged ions and can be further divided into two groups, the sealing claudins and the pore-forming claudins [review in (2)]. For example, claudin-1 (CLDN1) is one of the sealing claudins important for maintaining structure (3) and is translocated and downregulated during proinflammatory cytokine- (4) and hypoxia- (5) induced barrier dysfunction. In contrast, claudin 2 (CLDN2), a member of pore-forming claudins, is responsible for creating water and sodium (Na^+^) ion channels across the polarized epithelium (6, 7) and its loss is associated with attenuated colitis development (8). Furthermore, members of the claudin family interact with TAMPs and CTX in order to maintain the structural integrity of TJs (9, 10), while individual TAMPS and CTX also act as anchors to various signaling molecules [review in (11)]. Overall, the TJ transmembrane proteins work together to regulate the passive transport of water and small ions, provide structural integrity to TJ and enhance signaling cascades.

Myosin light chain (MLC) is part of non-muscle myosin (NMM) heterohexamer which forms the actomyosin ring present at the cell periphery and linked to the TJ protein complex [review in (12, 13)]. In general, contraction of actin-myosin rings in individual intestinal epithelial cells contributes to widening of intercellular spaces and subsequently allowing macromolecules to passively pass through paracellular space of the intestinal epithelium (leak pathway), and thus compromising intestinal barrier function. During intestinal inflammation, stimulation from proinflammatory cytokines on intestinal epithelium activates myosin light chain kinase (MLCK) and promotes phosphorylation of MLC2 (14), which leads to contraction of actomyosin ring (15) and reduced actin anchoring to TJ protein complex (16), resulting in a reduction in intestinal epithelial barrier function.

Lastly, in addition to the passive transport of ions, water and macromolecules through paracellular space, charged ions and macromolecules are also actively transported through epithelial cells from lumen through various ion transporters present in both apical and basolateral sides of intestinal epithelial cells. Intestinal epithelial Na^+^ ion transporters, such as, the sodium-hydrogen exchanger/solute carrier family 9 member 3 (SLC9A3), Na^+^-glucose cotransporter and Na^+^/K^+^ transporting ATPase, contribute to the active transport of Na^+^ ions across colonic epithelium [review in (17)]. Coincidently, activities of SLC9A3 and Na+-glucose cotransporter have been shown to affect electrical potential and MLC activation across intestinal epithelial cells (18, 19). Taken together, the flow of ions, water and nutrients across paracellular space is governed by intricate expression and interactions between TJ transmembrane proteins, MLC phosphorylation and ions transporters present on intestinal epithelial cells.

In patients with Inflammatory Bowel Diseases (IBD), a compromised barrier is often associated with recurrent and relapsing intestinal inflammation [review in (1, 20)]. Our previous studies have identified aftiphilin (AFTPH) to be downregulated during active colonic inflammation in colonic tissues from patients with ulcerative colitis (UC) and experimental colitis mouse models (21). Since previous studies have suggested that AFTPH is a binding partner of non-muscle myosin 2 (NMMII) (22, 23), we hypothesized that AFTPH might interact with components regulating epithelial permeability in colonic epithelial cells. In this study, we examined transepithelial electrical resistance (TEER), Na+ ion flux, expression of TJ transmembrane protein, MLCK activity and actin dynamics in intestinal epithelial cells in the presence and absence of AFTPH and showed that AFTPH deficiency increased epithelial cell permeability potentially by increasing MLCK activity and interfering with actin polymerization.

## Results

### Aftiphilin Downregulation Compromises Intestinal Epithelial Barrier Function *In Vitro*

**Figure 1:**
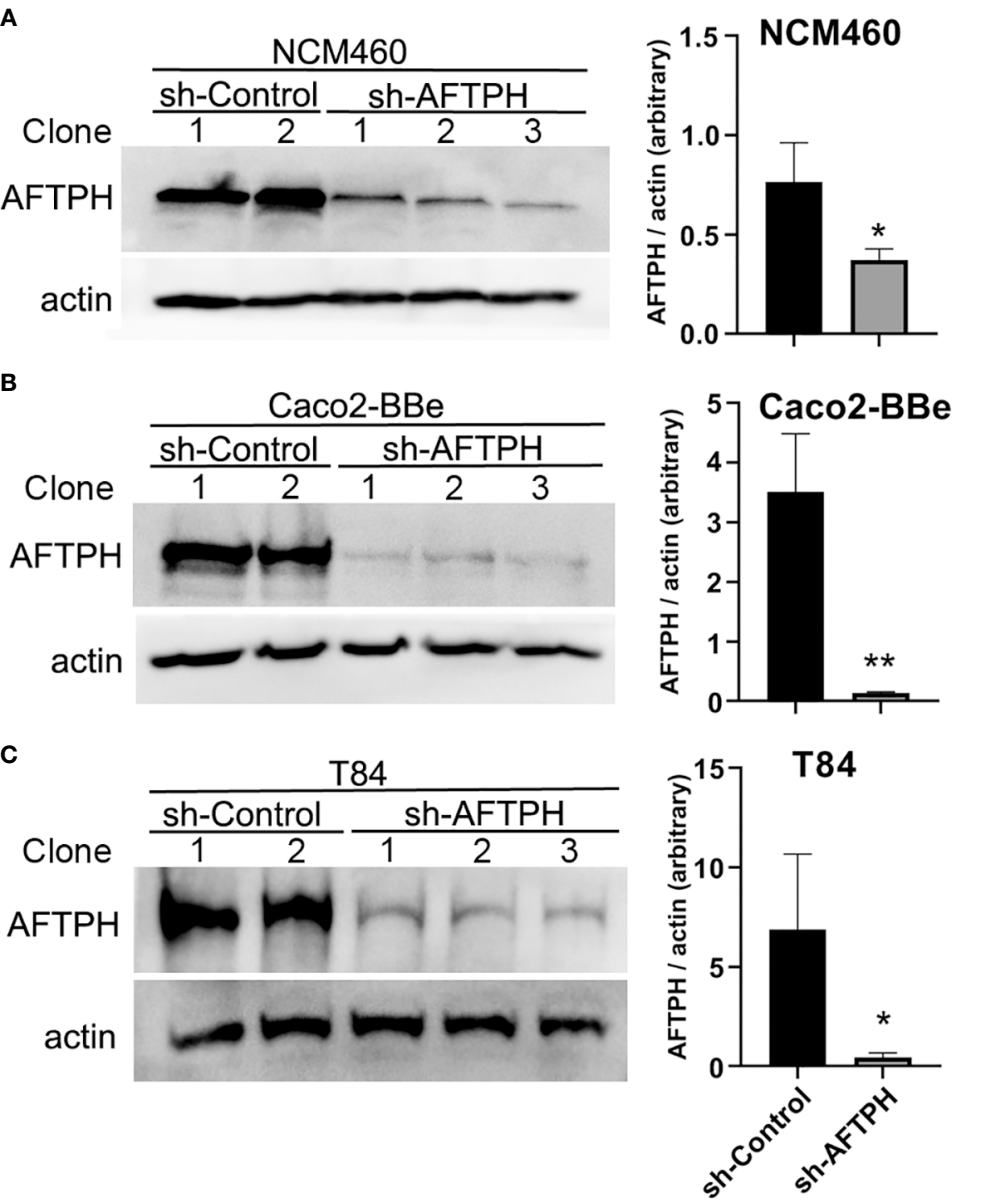
AFTPH expression is significantly reduced in colonic epithelial cell lines transduced with recombinant lentivirus expressing sh-AFTPH. Control recombinant lentivirus and recombinant lentivirus carrying sh-AFTPH were used to transduced 3 cell lines, **(A)** NCM460 (sh-control = 2, sh-AFTPH = 3), **(B)** Caco2-BBe (control = 2, sh-AFTPH = 3) and **(C)** T84, transduced with recombinant lentivirus expressing sh-AFTPH (control = 2, sh-AFTPH = 3). Individual clones were generated from sh-control and sh-AFTPH transduction. Expression of AFTPH in different clones was examined using Western Blot and densitometric analysis. Mean± SEM. *p<0.05, **p<0.01.

**Figure 2:**
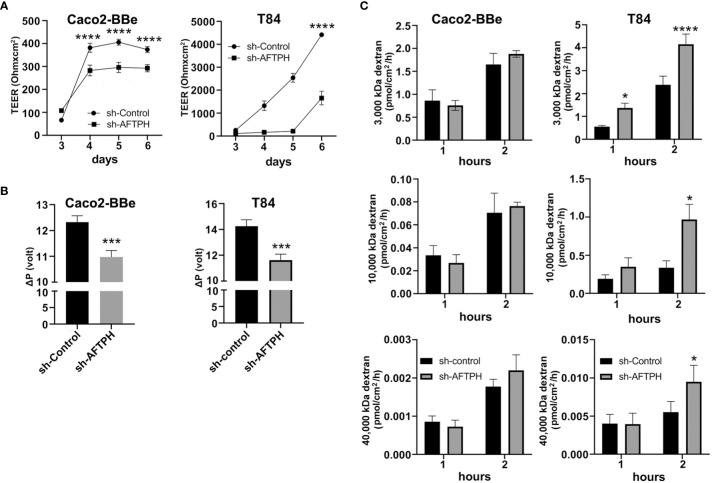
AFTPH gene-silencing significantly increases colonic epithelial permeability in Caco2-BBE and T84 monolayers. **(A)** Transduced sh-control and sh-AFTPH clones were plated on transwells on day 0 and allowed to polarize. The transepithelial electrical resistance (TEER) of intestinal epithelia derived from each clone were repeatedly measured by volt-ohm meter on each day from 3 to 6 after plating. Mean± SEM (n = 3). ****p < 0.0001 **(B)** Na^+^ ion permeability was measured by dilution potential assay as described in the Methods at day 6. Mean± SEM. (n=3) ***p<0.005. **(C)** Dextran of size 3000, 10,000, 40,000 M.W. was incubated with polarized sh-control and sh-AFTPH Cac2-BBe and T84 clones in dextran permeability assay as described in Methods at day 6 and measured at 1 and 2 hours after the start of the experiment. Mean± SEM. (n=3). *p<0.05, ****p<0.001.

According to The Human Protein Atlas project (https://www.proteinatlas.org/ENSG00000119844-AFTPH/cell) and other previous studies (23–26), AFTPH is expressed in most of the major cell types in the gut, such as, endothelial cells, immune cells, fibroblasts and neurons, in addition to our findings on colonic epithelial cells (21). Since compromised intestinal epithelial permeability is a hallmark of ulcerative colitis, here, we hypothesize that reduced AFTPH expression has a direct role in increasing paracellular permeability of colonic epithelial monolayer. To accomplish this, we transduced three different colonic epithelial cell lines with shRNA containing lentiviruses: NCM460, Caco2-BBe & T84. NCM460 cells are non-transformed colonic epithelial cells derived from normal human colonic mucosa (27). Caco2-BBe and T84 cells are both intestinal epithelial cell lines derived from colorectal adenocarcinoma and are well-established cell models for epithelial monolayer formation, closely resembling human colonic epithelium (28). As shown in **Figure 1**, NCM460, Caco2-BBE and T84 cell lines transduced with the AFTPH-targeting shRNA have 51.3 ± 7.29%, 96.0 ± 0.51% and 93.6 ± 3.29% reduction in AFTPH protein levels, when compared to same cell lines transduced with scramble shRNA, respectively (*p*<0.05, *p*<0.01, *p*<0.05). We then tested the formation and maintenance of permeability, which can be represented by TEER, in AFTPH gene-silenced Caco2-BBe and T84 monolayers. As shown in **Figure 2A**, AFTPH-deficient Caco2-BBe developed significantly lower TEER, while AFTPH deficiency significantly delayed the development of TEER in T84 monolayers. Since TEER is a measurement of overall ion flow through intestinal epithelium *in vitro*, we next examined Na^+^ ion and dextran permeability in Caco2-BBe and T84 monolayers gene-silenced with AFTPH and their control counterparts, as a representation of the flux of small, charged ions and uncharged molecules. Our results showed that, in both Caco2-BBe and T84 cells, AFTPH gene-silencing not only significantly lowered TEER (**Figure 2A**), but the dilution potential of Na^+^ ions was also lowered (12.3 ± 0.25 mV *vs*. 10.9 ± 0.15 mV; and 14.6 ± 0.64 mV *vs*. 11.4 ± 0.32 mV, *p*=0.0003), when compared to control cells (**Figure 2B**). Interestingly, with regards to flux of 3000, 10,000 and 40,000 M.W. dextran passing across the epithelial monolayers of both cell lines, our results showed differences between Caco2-BBE and T84 gene-silenced cells. In Caco2-BBe cells, AFTPH gene-silencing did not show altered permeability to dextran of all molecular weights (**Figure 2C**). In contrast, AFTPH-deficient T84 monolayers showed increased permeability to dextran of 3,000 M.W. by 1.7 ± 0.18 fold (*p*<0.0001), 10,000 M.W. by 2.9 ± 0.59 fold (*p*<0.05) and 40,000 M.W by 1.7 ± 0.39 fold (*p*<0.05) at 2 hours after incubation. Taken together, AFTPH deficiency increased epithelial permeability, potentially by increasing permeability to Na^+^ ions and, to a lesser extent, large molecules in colonic epithelial monolayer.

### Occludin Expression Is Modulated by AFTPH Gene-Silencing in Intestinal Epithelial Monolayers

**Figure 3:**
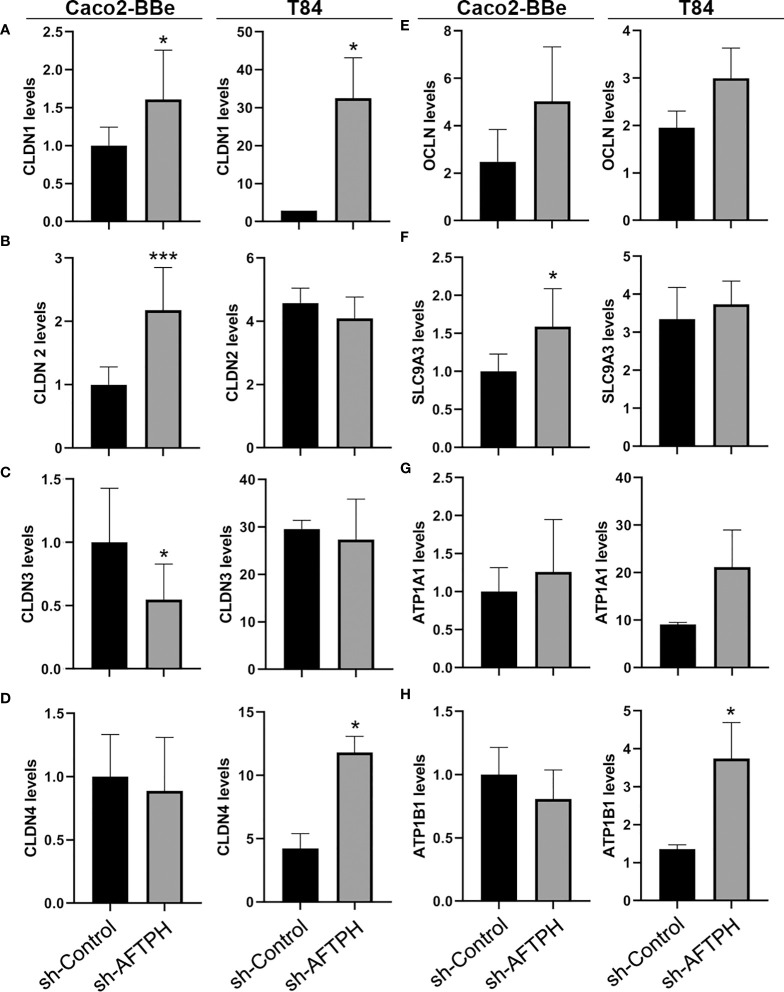
AFTPH deficiency does not uniformly downregulate mRNA levels of tight junction proteins and Na^+^ ion transporters in Caco2-BBe and T84 monolayers except CLDN1. After extracting RNA from polarized cells, RT-qPCR was performed to determine the gene expression of **(A–D)** CLDN1, -2, -3, -4; **(E)** OCLN, **(F)** SLC9A3, **(G)** ATP1A1 and **(H)** ATP1B1 in Caco2-BBE and T84 cells using qPCR. Mean± SEM. (n=3). *p<0.05, ***p<0.005.

Since Na^+^ ion flow is commonly elevated in both AFTPH-deficient cell lines, we next tested the role for AFTPH in the two major mechanisms for regulating paracellular ion flow, active ion transport (through transcellular Na^+^ ion transporters) and passive ion flow (pore pathway, through TJ proteins). We first examined gene expression of multiple claudins and occludin (OCLN) and discovered that AFTPH gene-silencing increased gene expression of claudin-1 (CLDN1) in both Caco2-BBe and T84 monolayers by 1.6 ± 0.08 fold (*p*=0.0259) and 11.2 ± 3.68 fold (*p*<0.05), respectively (**Figure 3A**). Expression levels of claudin-2, -3 and -4 (CLDN2, CLDN3, CLDN4) were also altered in both Caco2-BBe and T84 monolayers. Specifically, significantly higher expression levels of CLDN2 and lower expression levels of CLDN3 were observed in AFTPH-deficient Caco2-BBe cells (*p*<0.005, *p*<0.05, respectively). However, CLDN2 and CLDN3 mRNA levels were not altered in sh-AFPTH-transduced T84 monolayers (**Figures 3B, C**). Alternately, CLDN4 expression was significantly upregulated in T84 monolayers stably transduced with sh-AFTPH (*p*<0.05, **Figure 3D**), but, in Caco2-BBe cells, AFTPH gene-silencing did not affect CLDN4 expression. Interestingly, AFTPH deficiency slightly increased OCLN mRNA in both Caco2-BBe and T84 monolayers, although no statistical significance was reached (**Figure 3E**). Next, we examined expression of Na^+^ ion membrane transporters. Our results showed that SLC9A3 expression was significantly increased only in Caco2-BBe cells (*p*<0.05, **Figure 3F**); and expression of Na^+^/K^+^ transporting ATPase subunit ATP1B1 was significantly upregulated in T84 cells but not Caco-2 cells (*p*<0.05, **Figure 3H**). ATP1A1, another Na^+^/K^+^ ATPase subunit, was not significantly altered in either cell line (**Figure 3G**). Overall, our gene expression analysis demonstrated that reduced AFTPH expression did not affect the overall gene expression of these genes except CLDN1 and OCLN in intestinal epithelial cells.

**Figure 4:**
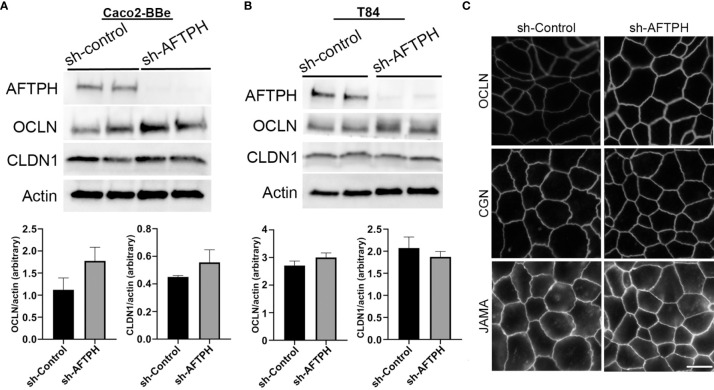
Reduced AFTPH expression increases Occludin membrane localization but does not significantly alter the structure of tight junction in T84 monolayers. **(A)** Caco2-BBe and **(B)** T84 sh-control and sh-AFTPH clones (n=2) were seeded and cultured in 6-well plate and harvested. Protein expression of AFTPH, OCLN, CLDN1 were examined using Western Blot, and compared as actin (loading control). Mean± SEM. **(C)** Structure of tight junction was visualized with antibodies against OCLN, CGN and JAMA using confocal microscopy in T84 cells. Time-exposure used in T84 monolayers transduced with sh-Control- and sh-AFTPH was specific to the antibodies used in labelling. This experiment was repeated 3 times. (Scale = 10 μm).

To determine whether the relative mRNA levels of TJ proteins correlate with protein alterations, protein expression of CLDN1 and OCLN in Caco2-BBe and T84 monolayers were further examined by western blot. Contrary to the results by qPCR, protein expression of CLDN1 was not significantly increased in both Caco2-BBe and T84 monolayers (**Figures 4A, B**). Similar to results from gene expression analysis, OCLN expression was slightly, but not significantly, increased in both cell lines (**Figures 4A, B**).

Another factor leading to increase in intestinal epithelial permeability is translocation of TJ proteins from the plasma membrane to the cytosol. To examine the plasma membrane localization of TJ associated proteins, immunofluorescence against OCLN, junctional adhesion molecule-A (JAM-A, a member of the CTX family) and cingulin (CGN) in acetone-fixed T84 monolayers was performed. OCLN and JAM-A are transmembrane proteins that form part of the TJ protein complex (9, 29, 30), while CGN is a linker protein connecting TJ protein complex and cytoskeleton (31–33) in intestinal epithelial cells. Results from immunofluorescence microscopy of T84 monolayers showed that AFTPH deficiency notably increased OCLN localization on the plasma membrane (**Figure 4C**). However, levels of plasma membrane-localized CGN and JAM-A remain similar in monolayers transduced with sh-control and sh-AFTPH (**Figure 4C**). These results suggest that enhanced permeability due to AFTPH deficiency is unlikely due to changes in the levels and localization of claudins, CGN, JAM-A and Na+ ion transporters but could be explained by stronger OCLN membrane localization.

### AFTPH Colocalizes With Myosin Light Chain Kinase and Regulates Its Activity in Intestinal Epithelial Monolayers

**Figure 5:**
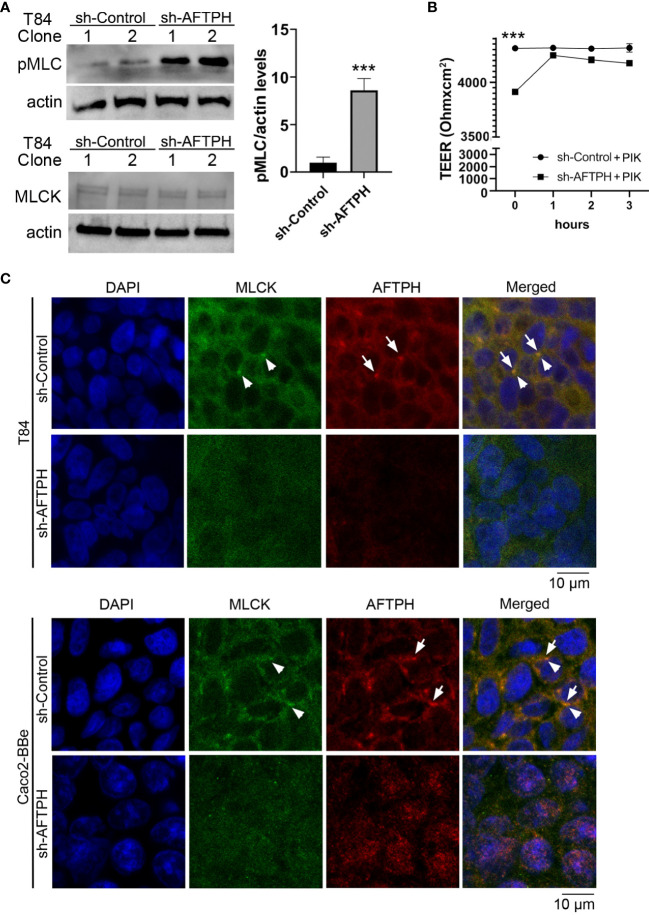
AFTPH colocalizes with MLCK and its deficiency increases MLCK activity. **(A)** Individual T84 sh-control (n=2) and sh-AFTPH (n=3) clones were cultured in 6-well plates and were harvested when confluent. Levels of phosphorylated MLC2 in different T84 clones was examined by Western blot. Levels of MLCK were analyzed in a separate experiment. Mean ± SEM. ***p<0.005 **(B)** Polarized T84 cells were treated with 5 μM PIK at day 6 and TEER was measured every hour (n=3). ***p<0.005 **(C)** Polarized T84 and Caco2-BBe cells were cultured for 6 days and colocalization of MLCK (arrowhead) and AFTPH (arrow) was examined by immunofluorescence microscopy. This experiment was repeated 3 times. (Scale = 10 μm).

As we observed that AFTPH regulated dextran flux in colonic epithelial T84 monolayers (**Figure 2**), we hypothesized that AFTPH deficiency promotes phosphorylation of MLC2, which would subsequently lead to contraction of peri-junctional actin-myosin rings and disrupted barrier function (15), potentially through upregulating MLCK-induced MLC2 phosphorylation at Ser 19. Interestingly, inactivation of MLCK activity has been shown to increase TEER of intestinal epithelial monolayers (15, 34). To test this hypothesis, we examined levels of MLC2 phosphorylation by western blot in individual sh-AFTPH-transduced T84 clones and their control counterparts and demonstrate that levels of pMLC2 were higher in AFTPH-gene silenced T84 cells by 8.8 ± 1.26 fold (*p*=0.0043, **Figure 5A**) despite similar MLCK expression, when compared to control cells. To determine if MLCK activity was required for the increased permeability in loss of function AFTPH cells, we blocked MLCK activity in T84 AFTPH-deficient and control cells by treating the cells with PIK, a pharmacological inhibitor of MLCK (35). Results from the measurement of TEER demonstrated that treatment with PIK reduced permeability of AFTPH-deficient T84 monolayers to levels similar to that of the control cells (**Figure 5B**), suggesting that increased overall permeability of sh-AFTPH-transduced T84 monolayers was partly due to elevated MLCK activity in these cells. To explore a potential mechanism leading to AFTPH interacting with MLCK in colonic epithelial cells, localization of AFTPH and MLCK were visualized by immunofluorescence confocal microscopy in differentiated Caco2-BBe and T84 cells. Interestingly, colocalization of AFTPH and MLCK was observed in control T84 and Caco2-BBe monolayers with Pearson’s correlation coefficient of 0.2719 and 0.34788, respectively (**Figure 5C**), suggesting that some of MLCK molecules were localized at intracellular vesicles or organelles (25). In contrast to control polarized epithelia, AFTPH-deficiency in both cell lines greatly reduced observed intracellular MLCK localization, despite similar MLCK expression in both cell lines and the increased MLCK activity in sh-AFTPH cells. In summary, our results suggest that AFTPH regulates colonic epithelial permeability partly through regulating MLCK localization and activity.

### AFTPH Deficiency Dysregulates Actin Assembly and Increases Microvilli Length at Brush Borders

**Figure 6:**
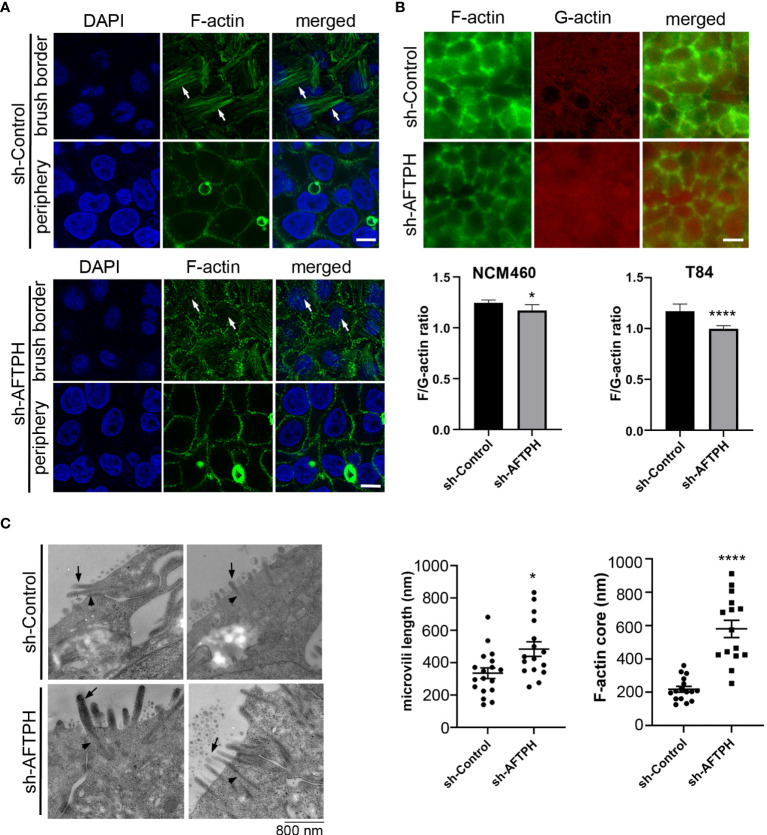
AFTPH depletion reduces actin polymerization and increases microvilli formation. **(A)** NCM460 sh-control and sh-AFTPH cells were seeded and fixed with 4% PFA. F-actin of at brush border and periphery were stained and visualized by confocal microscopy. This experiment was repeated 3 times. (Scale = 10 μm) **(B)** F-actin at peri-junctional actomyosin ring and G-actin monomer in polarized T84 sh-control and sh-AFTPH monolayers were stained and visualized by fluorescence microscopy. (Scale = 10 μm). NCM460 and T84 sh-control and sh-AFTPH cells were cultured in 96-well plates. F- and G- actin were stained and a fluorimeter was used to measure their intensity. F/G-actin ratios were then calculated. Mean ± SEM. (n=10). **(C)** Polarized T84 sh-control and sh-AFTPH monolayers were cultured and fixed as described in the Methods. Electron microscopy showing microvilli (arrow) and F-actin core (arrowhead) present in polarized T84 sh-control and sh-AFTPH monolayers. (Scale = 800 nm) Length of microvilli and F-actin core were measured using Image J. Mean± SEM. *p<0.05, ****p<0.001.

Since results from this study suggested that AFTPH may regulate activities of MLCK, which has two actin-binding domains, and also closely interact with non-muscle myosin (NMM) (22, 23), an actin-based motor protein; we next examined actin assembly at brush borders and the cell periphery by fluorescence microscopy after AFTPH deficiency in NCM460 cells. NCM460 cells was first studied in this assay since they are non-transformed cells (27), closely resembling normal mucosa epithelial cells. Filamentous (F-) actin in NCM460 cells at brush borders was significantly dysregulated upon AFTPH depletion, although F-actin at the cell periphery was not altered by the absence of AFTPH (**Figure 6A**). We then labelled F-actin and G-actin individually in control and AFTPH-deficient NMC460 and T84 cells and measured overall F-/G-actin ratio. Results from fluorescence spectrometry revealed that AFTPH deficiency significantly lowered F/G-actin ratio in NCM460 and T84 cells (*p*<0.05, *p*<0.0001, respectively), while the individual measurements of F- and G-actin were not different in the presence or absence of AFTPH (**Figure 6B**). In addition, confocal microscopy of T84 monolayers also showed no differences in peri-junctional actomyosin rings under AFTPH deficiency (**Figure 6B**). Lastly, we also studied the morphology of microvilli in control and AFTPH-deficient polarized T84 epithelia. T84 monolayers were fixed and imaged by transmission electron microscopy and interestingly, we found that microvilli were more abundant and longer on cell surface of AFTPH-deficient T84 cells (**Figure 6C**, as indicated by arrows). A close look at the electron micrographs suggested that F-actin cores in terminal webs of AFTPH-deficient T84 monolayers were also longer than those in control monolayers (**Figure 6C**, as indicated by arrowheads). Further statistical analysis showed that sh-AFTPH monolayers have longer microvilli and F-actin core (*p*<0.05, *p*<0.0001, respectively). Taken together, results from our study suggested that AFTPH colocalized with MLCK and regulated its activity on MLC and actin, subsequently affecting intestinal barrier function and microvilli growth.

## Discussion

Previous studies have demonstrated that AFTPH plays a role in exocytosis (26), intracellular trafficking (23) and receptor recycling (25) in various cell types. While our studies have demonstrated reduced AFTPH levels in colonic tissues during colonic inflammation, potentially promoting proinflammatory signaling in colonic epithelial cells (21); we examined the potential role and molecular mechanism of AFTPH in regulating colonic epithelial permeability *in vitro* in our present study. Our results demonstrate that reduced AFTPH levels in colonic epithelial cells led to impaired TEER, increased Na^+^ ion flow, and increased dextran permeability. Notably, our experimental evidence strongly suggested that differences in TEER between control and AFTPH-deficient colonic epithelial monolayers were largely due to differences in Na^+^ ion flow (**Figure 2**). However, no candidates from the claudin family, including claudin-2, which can form Na^+^ ion channel [review in (2, 7)], and Na^+^ ion transporters were commonly dysregulated in both Caco2-BBE and T84 AFTPH-silenced monolayers (**Figures 3**, **4**). On the other hand, results from confocal microscopy suggested a novel finding that MLCK, in which its kinase activity is essential to contraction of peri-junctional actomyosin ring (15), is colocalized with AFTPH in both T84 and Caco2-BBe cells intracellularly (**Figure 5**). Furthermore, examination of the molecular alterations after AFTPH gene-silencing showed that MLCK activity was increased, while actin polymerization was reduced in AFTPH-deficient T84 cells (**Figures 5**, **6**). As for limitations in this study, we observed increased MLCK activity but reduced colocalization with AFTPH in sh-AFTPH cells, which appeared to be contradictory. Furthermore, only cell surface localization of claudins, and other TJ proteins under AFTPH deficiency was examined, the intracellular localization of these proteins could not be determined due to acetone fixation of the cells (36). Thus limiting our understanding of AFTPH function on intracellular trafficking of these proteins, which may be crucial to maintaining intestinal permeability *in vitro*. Further examination of intracellular trafficking of TJ proteins and MLCK may provide further insights to AFTPH function.

In this study, our results demonstrate that AFTPH deficiency leads to increased MLCK activity (**Figure 5**), which coincides with increased Na^+^ ion flux in the presence of a transepithelial Na^+^ ion gradient (**Figure 2B**). Furthermore, inhibition of MLCK activity reverses increased intestinal epithelial permeability (TEER) caused by elevated MLCK activity *in vitro* (**Figure 5**), similar to results observed in previous studies (15, 34, 35). We hypothesize that increased MLCK activation in AFTPH-depleted colonic epithelial monolayers promotes contraction of peri-junctional actomyosin ring (15) and/or destabilization of TJ protein complex (34); thus, in the presence of transepithelial Na^+^ gradient (**Figure 2C**), increased number of Na^+^ ions passes through transcellular space across colonic epithelium and leads to reduction in Na^+^ ion dilution potential when compared to control epithelial monolayers. Interestingly, AFTPH deficiency-induced MLCK activation did not result in a uniform increase in transepithelial dextran flux in both Caco2-BBe and T84 monolayers (**Figures 2**). Previous studies have also shown that activation of MLCK may lead to differential regulation of ion and macromolecules flux under different physiological stimuli. The most well-studied examples are regulation of paracellular flux during Na^+^-glucose cotransport and stimulation from tumor necrosis factor (TNF) through MLCK activation. During Na^+^-glucose cotransport, sodium-dependent glucose transporter (SGLT), located at the brush-borders, is activated and this transactivates MLCK in intestinal epithelial cells (18, 37). Increased pMLC2 after loss of AFTPH present at peri-junctional actomyosin ring is the result from activation of MLCK (18), potentially induces contraction of peri-junctional actomyosin ring (15) and thus, increases paracellular space in intestinal epithelium. Together with activation Na^+^ ion transporters, SLC9A3 (NHE3) (19) and Na^+^/K^+^ transporting ATPase, a Na^+^ ion concentration gradient is generated across colonic epithelium, allowing increased flow of water, along with small solute (size: <4Å), across epithelium through paracellular space (38). In TNF-induced paracellular flux, TNF, together with other proinflammatory cytokines, increases intestinal epithelial permeability (14, 39) through activation of MLCK (14). Interestingly, TNF-induced MLCK activation allows macromolecules flux (e.g. sizes of dextran: > 14Å) across intestinal epithelium (14, 39) and is the major mechanism promoting TNF-induced diarrhea (40). The detailed mechanisms leading to these two different forms of MLCK-dependent paracellular flux are not well understood.

**Figure 7:**
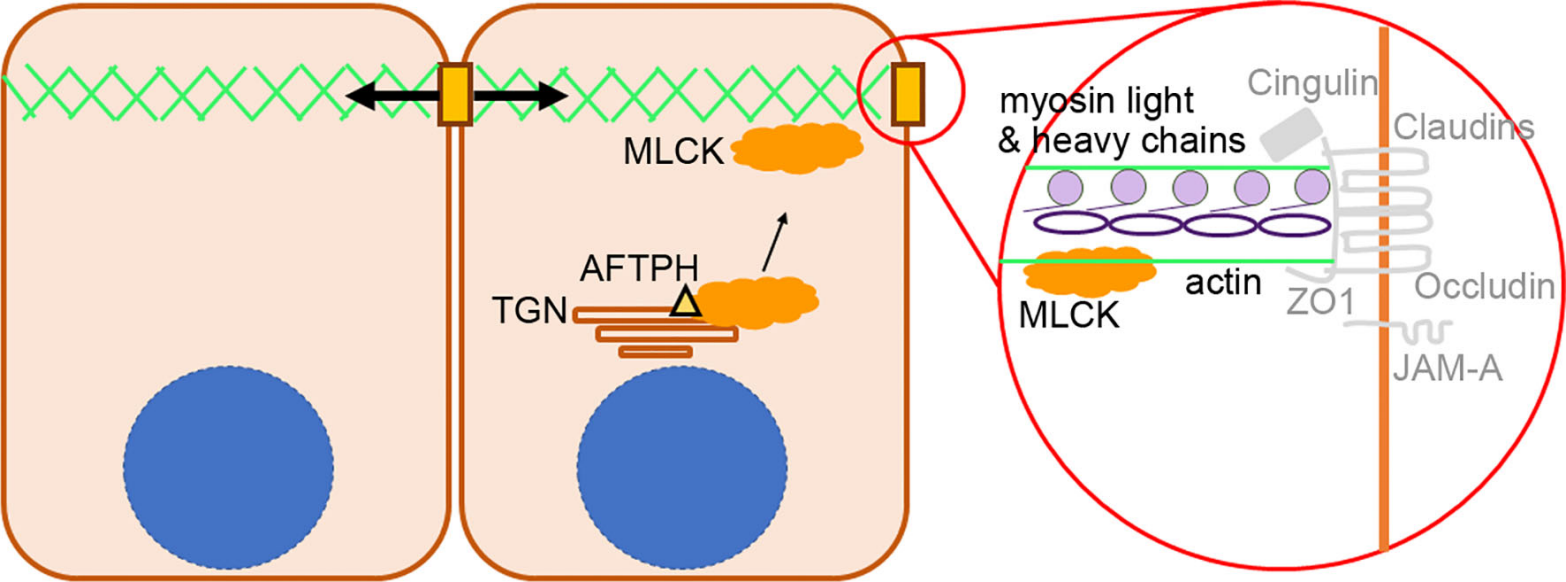
Proposed role of AFTPH in facilitating translocation of MLCK. A diagram showing the proposed role of AFTPH in transporting MLCK from trans-golgi network (TGN) to peri-junctional actomyosin ring to promote the phosphorylation of MLC.

On the other hand, our findings on colocalization of MLCK and AFTPH provided novel insights on regulation of MLCK activity. There are 2 isoforms of MLCKs expressed in mammalian muscle and non-muscle cells, namely long MLCK isoform (220 kDa) and short MLCK isoform (130 kDa) (41). In biopsies taken from human small intestine, long MLCK isoform is localized at the cytoplasm adjacent to brush borders (42). In contrast, in polarized Caco2-BBe cells, long MLCK isoforms were localized in the cytoplasm, but was translocated to peri-junctional actomyosin ring upon TNF stimulation (42). Our results provided evidence supporting the notion that MLCK can be localized in cytosol with AFTPH, potentially at TGN or early endosomes (25) in polarized Caco2-BBe and T84 monolayers (**Figure 5**). In addition, our novel findings on intracellular MLCK/AFTPH colocalization may have important implications in revealing mechanisms regulating of MLCK activities (**Figure 7**). Analysis of amino acid sequences suggests that both MLCK isoforms comprise of a catalytic domain, 2 actin-binding domains, 2 calmodulin-binding domains and 1 myosin-binding domain [review in (43), **Figure 7**]. This is evident in co-localization studies in fibroblasts revealing that the long MLCK isoform colocalizes with F-actin, while short MLCK isoform is localized with non-muscle myosin 2A (NMMIIA) (41, 44). Non-muscle myosin 2 (NMMIIs) belong to myosin superfamily and are structurally grouped as heterohexamers [review in (45, 46)]. As shown in **Figure 7**, NMMIIs are comprised of 2 heavy chains which possess enzymatic activities, which drive ATP-dependent actin filament movement; 2 essential light chains which stabilize the heavy chain structure; and regulatory light chain (also called MLC2) which is the target of MLCK catalytic domain and regulates NMMs activity in actomyosin complex as described (37, 40, 44, 47). Previous studies demonstrated that the heavy chain of NMMIIs and AFTPH have similar functions in intracellular trafficking in polarized epithelial cells (22, 24, 25, 48); while NMMII heterohexamer is a binding partner of AFTPH in HeLa cells (23). Therefore, our results highly suggest that AFTPH is essential in mediating the direct interaction between MLCK and NMMII/MLC2 and this interaction, subsequently, affect the integrity of intestinal barrier (**Figure 7**).

There is an implication that the AFTPH/MLCK/NMMII network may also explain changes in Occludin expression in AFTPH-depleted cells. Similar to the results of the present study (**Figure 4**), depletion of NMMIIA also results in functional defective, but structurally normal cellular junctions in intestinal epithelial monolayers (49). In particular, although localization of Occludin remains similar under NMMIIA depletion, upon destabilization of cellular junctions induced by calcium depletion, the majority of Occludin remains in cell-cell junctions in NMMIIA-depleted monolayers, while this phenomenon was not observed in control monolayers (49). Although the roles of Occludin in regulating intestinal epithelial permeability and tight junction structural integrity remains to be defined (9, 29, 50–52), the results of our study suggest that potential AFTPH-NMMIIA interaction may affect Occludin intracellular trafficking, which may have important implications to Occludin translocation and formation of TJ protein complex (53–55).

Lastly, in addition to dysregulated intestinal permeability, we also observed lower polymerization of actin monomers, disorganized F-actin filaments and increased number and length of microvilli in AFTPH-deficient intestinal monolayers (**Figure 6**). A recent study by Chinowsky et al. demonstrated that NMMII is involved in actin polymerization/depolymerization and growth of epithelial microvilli (56). Thus, AFTPH-dependent actin polymerization and microvilli growth may also be associated with NMMII function in intestinal epithelial cells. In conclusion, our results highly suggest that AFTPH-MLCK interaction play an essential role in regulating the development and stability of cellular junctions and peri-junctional actomyosin ring in intestinal epithelial cells, potentially in collaboration with NMMIIA. Further investigation on the interactions between AFTPH, NMMIIA, MLCK may reveal novel insights to mechanisms regulating development and stability of cellular junctions in intestinal epithelium (**Figure 7**).

## Experimental Procedures

### Cell Culture

Colonic epithelial NCM460 cells were maintained with M3D media (INCELL Inc.); Caco2-BBE (ATCC CRL-2102) and T84 cells (ATCC CCL-248) were maintained with DMEM and DMEM/F:12. Base media was supplemented with 10% fetal bovine serum (FBS) and 1% Penicillin/Streptomycin (ThermoFisher Inc., Calrsbad, CA). Stable AFTPH knock-down clones were created by transducing NCM460, Caco2-BBE and T84 cells with recombinant lentivirus (Dharmacon ™ SMARTvector ™) expressing short hairpin RNAs (shRNAs) against AFTPH (Nbla10388, Horizon Discovery) and selected to create stable knock-down clones. Control clones were created by transducing the colonic epithelial cell lines with recombinant lentivirus expressing short hairpin with scramble sequence. Individual transduced clones from control shRNA and sh-AFTPH transduction were generated and levels of AFTPH were verified using PCR and western blot analysis. 2 control clones and 3 sh-AFTPH clones were used in subsequent experiments.

### Measurement of Transepithelial Electrical Resistance

To measure transepithelial electrical resistance (TEER), Caco2-BBE cells (1x10^5^ cells) or T84 cells (1.5 x 10^5^ cells) were seeded on semi-permeable supports (0.33 cm^2^, pore size: 0.4um Corning) and cultured for 4-7 days. A Millicell ERS-2 epithelial volt-ohm meter (Millipore) was used to measure TEER of each monolayer daily from day 3 to the end of the experiment. In the experiments described below, Caco2-BBE and T84 cells carrying sh-control had a TEER of at least 400 mΩ·cm^2^ and 2000 mΩ·cm^2^, respectively.

For dilution potential experiments, cells were seeded and cultured on the semi-permeable supports for 4-7 days as described previously by Buchert et al. (57). In brief, background transepithelial voltage (TEV, P_bkgd_) was measured by recording the voltage difference between apical and basal chamber of a permeable support without cells. Culture medium was replaced with Buffer A (120 mM NaCl, 10 mM HEPES, pH 7.4, 5 mM KCl, 10 mM NaHCO_3_, 1.2 mM CaCl_2_, and 1 mM MgSO_4_) in both apical and basal chamber and TEV in Solution A (P_A_) was measured. Then, a second TEV measurement (P_B_) was performed immediately after Buffer A in apical chamber was replaced by Buffer B (60 mM NaCl, 120 mM mannitol, 10 mM HEPES, pH 7.4, 5 mM KCl, 10 mM NaHCO_3_, 1.2 mM CaCl_2_, and 1 mM MgSO_4_). Dilution potential (ΔP) was calculated as follows: (P_A_ - P_bkgd_) - P_B_.

To measure dextran permeability, dextran polymers tagged with Alexa Fluor™ 680 (3,000, 10,000 MW, Thermofisher) and dextran tagged with fluorescein (40,000 MW, Sigma Aldrich) were resuspended in 100 μl HBSS (4 mg/ml, 5 mg/ml, 25 mg/ml, respectively) and placed in the apical chamber of the Transwell inserts. Samples from basolateral chambers were collected at 1 and 2 hours after the start of the experiment and signals from the samples were read by a fluorimeter (BioTek Instruments, Winooski, VT, USA) to determine the amount of dextran passing through the epithelial monolayers.

### Filamentous-/Globular (F/G) -Actin Ratio

Stable NCM460 and T84 cell lines carrying sh-AFTPH and sh-control RNAs (23,000 cells/well, n=10) were seeded in 96-well black-walled microplates for fluorescence-based assays (Molecular Probes^®^, ThermoFisher). The cells were fixed 24 hours after seeding in a 4% buffered formaldehyde solution, pH 6.9 (Millipore Sigma) for 20 minutes at room temperature. The fixed cells were then permeabilized with 0.1% Triton-X 100 in PBS and stained with ActinGreen 488 ReadyProbes and Dexyribonuclease 1, Alexa Fluor ™ 594 conjugate (ThermoFisher) at room temperature for 20 minutes. The stained cells were then washed with 3 times with 0.1% Tween-20 in PBS. The signals from the cells were then detected by a fluorimeter (BioTek). F/G-actin ratio of individual wells were calculated as follows: OD_488(cell)_ - OD_488(background)_/OD_594(cell)_ – OD _594(background)_.

### Fluorescence Microscopy

To visualize F-actin in NCM460 cells, cells were fixed and stained as described above, using ActinGreen 488 ReadyProbes. For TJ proteins, Caco2-BBe and T84 epithelial monolayers were cultured on permeable support and fixed with pre-cooled acetone for 20 minutes at -20°C. The cells were then washed with PBS and stained with antibodies against tight junction proteins, such as, JAM-A (SAB4200468, Millipore Sigma), Cingulin (117796, Abcam), AFTPH (122239, Abcam), Occludin (#71-1500, Invitrogen), MLCK (# PA5-79716, Invitrogen) overnight at 4°C. After washing with 0.1% Tween-20 in PBS, the cells were stained with appropriate secondary antibodies. To visualize actin monomers and filaments in NCM460 and polarized T84 cells, cells were fixed and stained as stated above. The localization of different tight junction proteins, actin filaments were imaged with a Zeiss LSM 510 Meta laser scanning confocal microscope using a Zeiss 63x Plan-Apo/1.4 oil immersion objective (numerical aperture 1.4).

### Transmission Electronic Microscopy

Polarized T84 monolayers were fixed in 2.5% glutaraldehyde and 2% formaldehyde in 0.1 M sodium phosphate buffer (PB) overnight at 4°C. After washing, samples were post-fixed in 1% osmium tetroxide in 0.1M PB and dehydrated through a graded series of ethanol concentrations. After infiltration with Eponate 12 resin, the samples were embedded in fresh Eponate 12 resin and polymerized at 60°C for 48 hours. Ultrathin sections of 77 nm thickness were prepared and placed on formvar carbon coated copper grids and stained with uranyl acetate and Reynolds’ lead citrate. The grids were examined using a JEOL 100CX transmission electron microscope at 60 kV and images were captured by an AMT digital camera (Advanced Microscopy Techniques Corporation, model XR611) at Electron Microscopy Core Facility, UCLA Brain Research Institute. The length of microvilli and F-actin core were measured using Image J.

### Quantitative RT-PCR

Total RNA from Caco2-BBe epithelial monolayers was isolated using standard TRIzol reagent protocol (Life Technologies, Carlsbad, CA). A High Capacity cDNA Reverse Transcription Kit (ThermoFisher) used to generate cDNA libraries from equal amounts of total RNA (500 ng). Quantitative RT-PCR (qRT-PCR) for various gene expression was performed using iTaq Universal SYBR Green Mix (Bio-Rad) as described previously (58). Specific primers against CLDN1, CLDN2, CLDN3, CLDN4 and OCLDN were purchased from Integrated DNA Technologies. The sequence of the primers is listed in **Table 1**.

**Table 1:** Nucleotide sequences of primers used for RT-PCR.

Gene	Forward primers	Reverse primers
CLDN1	5’-CCGTTGGCATGAAGTGTATG-3’	5’-AGCCAGACCTGCAAGAAGAA-3’
CLDN2	5′-TGGCCTCTCTTGGCCTCCAACTTGT-3	5′-TTGACCAGGCCTTGGAGAGCTC-3′
CLDN3	5′-CATCACGTCGCAGAACATCT-3′	5′-AGCAGCGAGTCGTACACCTT-3′
CLDN4	5’-AAGGTGTACGACTCGCTGCT-3’	5’-CTTTCATCCTCCAGGCAGTT-3’
OCLN	5’-TTTGTGGGACAAGGAACACA-3’	5’-TAGTCAGATGGGGGTGAAGG-3’
ATP1A1	5′-AGGAATTCGGTCTTCCAGCA-3′	5′-ACCACCAGGTAGGTTTGAGG-3′
ATP1B1	5′-GAGGGCAAACCGTGCATTAT-3′	5′-TCTTCATCTCGCTTGCCAGT-3′
SLC9A3	5′-CTGAAGGTGAAGAGGAGCGA-3′	5′-GTGGGACCACTTGTCTCTGA -3′
MYLC	5′-TCACCTGGCTCAAGGGAAAT-3′	5′-TCCCACGTCATCTTGGTTGA -3′

### Western Blots

Confluent T84 epithelial monolayers were lysed in RIPA buffer supplemented with protease inhibitors (ThermoFisher) and denatured at 95°C for 5 minutes. Samples were added to a 4% to 20% SDS-containing polyacrylamide gel (Bio-Rad Laboratories), and a Trans-Blot Turbo system (Bio-Rad Laboratories) was used to transfer proteins to PVDF membranes. Membranes were blocked (phosphate-buffered saline, 5% nonfat dry milk, 0.05% Tween-20) and probed with antibodies followed by corresponding horseradish peroxidase–labeled secondary antibodies. A ChemiDoc Touch Imager and Clarity enhanced chemiluminescence kit were used to develop the blots (Bio-Rad Laboratories). Antibodies used include Claudin 1 (SAB4200462, Sigma Aldrich), Claudin 2 (ab53032, Abcam), Claudin 3 (SAB4500435, Sigma Aldrich), Occludin (#71-1500, Invitrogen), pMLC2 (3671, Cell Signaling). Data are represented by cropped images from the original membranes.

### Statistical Analysis

Unless otherwise stated, all experiments were performed three independent times. Graphpad prism was used to analyze all experimental data. A Student *t-test* was used to compare the statistical significance between control and sh-AFTPH groups unless otherwise stated. For TEER measurements over time, 2-way ANOVA was used to analyze the difference between control and sh-AFTPH groups over time. Samples run in triplicate and data represent mean ± SEM.

## Data Availability Statement

The original contributions presented in the study are included in the article/supplementary material. Further inquiries can be directed to the corresponding authors.

## Author Contributions

All authors contributed to the article and approved the submitted version.

## Funding

Supported by R01 NIH DK60729, DK47373, CURE : DDRC P30 DK 41301, NIH DK110003 (CP); Crohn's and Colitis Foundation #508773 (IKML); the Blinder Research Foundation for Crohn’s Disease (CP), and the Eli and Edythe Broad Chair (CP).

## Conflict of Interest

The authors declare that the research was conducted in the absence of any commercial or financial relationships that could be construed as a potential conflict of interest.

## Publisher’s Note

All claims expressed in this article are solely those of the authors and do not necessarily represent those of their affiliated organizations, or those of the publisher, the editors and the reviewers. Any product that may be evaluated in this article, or claim that may be made by its manufacturer, is not guaranteed or endorsed by the publisher.

## Abbreviations

AFTPH, Aftiphilin; MLCK, myosin light chain kinase; TJ, tight junction; TEER, transepithelial electric resistance; TGN, trans-golgi network.
